# Supplementary material for: Outcome measures for children with speech sound disorder: an umbrella review protocol
Source: BMJ Open. 2023 Feb 16;13(2):e068945. doi: 10.1136/bmjopen-2022-068945 (PMC9936291; doi:10.1136/bmjopen-2022-068945)
Supplement: Author's manuscript [file bmjopen-2022-068945.draft_revisions.pdf]

BMJ Open is committed to open peer review. As part of this commitment we make the peer review history of every article we publish publicly available.

When an article is published we post the peer reviewers' comments and the authors' responses online. We also post the versions of the paper that were used during peer review. These are the versions that the peer review comments apply to.

The versions of the paper that follow are the versions that were submitted during the peer review process. They are not the versions of record or the final published versions. They should not be cited or distributed as the published version of this manuscript.

BMJ Open is an open access journal and the full, final, typeset and author-corrected version of record of the manuscript is available on our site with no access controls, subscription charges or pay-per-view fees (http://bmjopen.bmj.com).

If you have any questions on BMJ Open's open peer review process please email info.bmjopen@bmj.com

BMJ Open

Outcome measures for Children with Speech Sound Disorder: An Umbrella Review Protocol

Journal:	*BMJ Open*
Manuscript ID	bmjopen-2022-068945
Article Type:	Protocol
Date Submitted by the Author:	06-Oct-2022
Complete List of Authors:	Harding, Sam; North Bristol NHS Trust, Bristol Speech and Language Therapy Research Unit; North Bristol NHS Trust, Research and Innoviation<br>Burr, Sam; North Bristol NHS Trust, Bristol Speech and Language Therapy Research Unit<br>Cleland, Joanne; University of Strathclyde, Psychological Sciences and Health<br>Stringer, Helen; Newcastle University<br>Wren, Yvonne; North Bristol NHS Trust, Bristol Speech & Language Therapy Research Unit; University of Bristol, Bristol Dental School
Keywords:	Protocols & guidelines < HEALTH SERVICES ADMINISTRATION & MANAGEMENT, Speech pathology < OTOLARYNGOLOGY, Health policy < HEALTH SERVICES ADMINISTRATION & MANAGEMENT

SCHOLARONE™
Manuscripts

Outcome measures for Children with Speech Sound Disorder: An Umbrella Review Protocol

Harding, S.A.[1,2], Burr, S. [1,2], Cleland, J.[3], Stringer, H.[4], Wren, Y.[1,5,6]

Corresponding Author:
Dr Sam Harding, Research Fellow
[1]Bristol Speech and Language Therapy Research Unit, North Bristol NHS Trust, Southmead Hospital, Bristol, BS10 5NB
[2]Research and Innovation North Bristol NHS Trust, Southmead Hospital, Bristol, BS10 5NB
Email: sharding.jb@gmail.com
ORCiD: 0000-0002-5870-2094

Authors:
Dr Sam Burr
[1]Bristol Speech and Language Therapy Research Unit, North Bristol NHS Trust, Southmead Hospital, Bristol, BS10 5NB
[2]North Bristol NHS Trust, Southmead Hospital, Bristol, BS10 5NB
Email: sam.burr@nbt.nhs.uk
ORCiD: 0000-0002-4177-2459

Dr Joanne Cleland
[3]Psychological Sciences and Health, Graham Hills Building, University of Strathclyde, Glasgow, Scotland
Email: joanne.cleland@strath.ac.uk
ORCiD: 0000-0002-0660-1646

Dr Helen Stringer
[4]King George VI Building, Newcastle University, Queen Victoria Road, Newcastle upon Tyne, NE1 7RU
Email: helen.stringer@ncl.ac.uk
ORCiD: 0000-0002-7470-2166

Dr Yvonne Wren
[1]Bristol Speech and Language Therapy Research Unit, North Bristol NHS Trust, Southmead Hospital, Bristol, BS10 5NB
[5] Cardiff School of Sport and Health Sciences, Cardiff Metropolitan University, Llandaff Campus, Western Avenue, Cardiff, Wales CF5 2YB
[6] Bristol Dental School, University of Bristol, Lower Maudlin St, Bristol, UK, BS1 2LY Email:
Email: Yvonne.Wren@Bristol.ac.uk
ORCiD: 0000-0002-1575-453X

Word Count: 2332

Key words: Umbrella review, protocol, speech sound disorder, assessment, intervention, outcomes

For peer review only - http://bmjopen.bmj.com/site/about/guidelines.xhtml

Outcome measures for Children with Speech Sound Disorder: An Umbrella Review Protocol

Abstract

Introduction: Speech sound disorder (SSD) describes a 'persistent difficulty with speech sound production that interferes with speech intelligibility or prevents verbal communication'. There is a need to establish which care pathways are most effective and efficient for children with SSD. Comparison of care pathways requires clearly defined, evidence-based, interventions and agreement on how to measure the outcomes. At present no list of assessments, interventions or outcomes exists.

The objective of this paper is to provide a rigorous and detailed protocol for an umbrella review of assessments, interventions, and outcomes which target SSD in children. The protocol details the development of a search strategy, and trial of an extraction tool.

Methods and Analyses: The umbrella review has been registered with PROSPERO (CRD42022316284). Papers included can use a review methodology of any sort, but must include children of any age, with a SSD of unknown origin. In accordance with the Joanna Briggs institute scoping review methods guidelines, an initial search of the Ovid Emcare and Ovid Medline databases was conducted. Following this a final search strategy for these databases were produced. A draft extraction form was developed.

Dissemination: Following the systematic development of an initial search strategy and extraction form, an umbrella review of this topic can take place. The development of a rigorous umbrella review protocol is essential in enhancing the transparency and reliability of the subsequent review. A pre-developed search strategy and trialling of an extraction form is a fundamental part of this process.

For peer review only - http://bmjopen.bmj.com/site/about/guidelines.xhtml

Introduction

Terminology and prevalence

Speech sound disorder (SSD) describes a 'persistent difficulty with speech sound production that interferes with speech intelligibility or prevents verbal communication'.[1] Prevalence is high, with estimates of 3.6% in children aged 4-8,[2-4] and upwards of 76,000 children referred to National Health Service (NHS) Speech and Language Therapy (SLT) services annually.[5] While a minority of children with SSD have clear aetiology (e.g., cleft palate, cerebral palsy, hearing impairment), in most cases, SSD has no identifiable cause and the evidence for intervention for this group is limited. Untreated, the impact of SSD is far reaching, leading to poor outcomes in education, employment and mental health.[6-9] NHS SLT is provided to children with SSD via a range of care pathways, typically defined by resource constraints, rather than robust evidence.

Implications for clinical practice

There is a need to establish which care pathways are most effective and efficient for children with SSD. Comparison of care pathways requires both clearly defined, evidence-based, interventions and agreement on how best to measure the outcomes of these interventions for children with SSD. However, a review of existing case notes of children treated for SSD were found to be too incomplete to compare pathways.[10] Additionally, it was found that the clinical case notes varied significantly between and within SLT services, thereby negating comparison. As a first step to determining which care pathways are most effective and efficient, this umbrella review will ask which assessment and outcome measures are commonly employed with children with SSD. Moreover, Morgan et al., suggests that there is a need for agreement on a national core outcome set (COS) for SSD.[11] A national consultation in 2018 identified the need to collect consistent data and recommended that NHS England support providers to collect data on the quality and outcomes of interventions (recommendation 4.5, p.29).[12]

Reviews to date

Evidence from systematic reviews and trials has shown that intervention is effective for the majority of children with SSD and that these children do not make progress without intervention.[13,14] However, studies have typically employed intervention protocols which are intense and are difficult to replicate in NHS SLT services.[15-17] Moreover, unlike research studies, clinical intervention takes place within care pathways which vary in terms of timing of intervention (pre-school, school age), delivery by speech and language therapists (SLTs) or assistants, number, frequency and duration of sessions, and involvement of parents or education staff, as well as the assessments and outcome measures used.

Previous research has identified that functional goals such as independence and improved social interaction are of greatest importance to parents,[17] while children listed improved speech alongside improved behaviour, schoolwork and skill at sports as well as making friends as important goals.[18,19] Preferred outcomes for pre-school children with SSD amongst SLTs have been identified as: intelligibility, social interaction and participation.[20] However, although a list of assessment tools (i.e., the tools used to obtain a speech sample) was compiled as part of this work,[20] it did not consider which specific analysis (e.g., percentage consonants correct, consonant inventory, error pattern analysis) is preferred to

For peer review only - http://bmjopen.bmj.com/site/about/guidelines.xhtml

measure the primary outcome or how this relates to functional domains (e.g., social interaction, participation, inclusion).

Review objective

The proposed umbrella review objective is to review the assessment and outcome measures used with children with SSD within speech and language therapy.

Review question

What assessment, interventions and outcomes are reported for children with SSD in health, social care and education?

Methods and Analysis

The review will be conducted in accordance with the Joanna Briggs Institute (JBI) methodology for umbrella reviews,[21] with an addition relating to quality appraisal where the AMSTAR tool will be used.[22] This is a literature review and therefore ethical approval is not required. The umbrella review has been registered with PROSPERO (CRD42022316284).

Patient and Public Involvement

As this is a review of published literature, there was no patient or public involvement in the development of this protocol.

Eligibility criteria

In line with the JBI guidance, the eligibility for included studies will be outlined according to population, phenomena of interest and context of data.[21] As this will be an umbrella review, the only papers retained for inclusion will be reviews. This can include any type of review e.g., systematic reviews of effectiveness, mixed methods, qualitative, scoping reviews.[23]

Population

The included population is children of any age with a diagnosis of SSD. Studies will not be excluded if the interventions include additional intervention targets (e.g., for receptive language). Children whose speech needs are associated with a biomedical condition with a known association with communication, such as sensorineural deafness, autistic spectrum condition or cleft lip and palate and neurological conditions (e.g., cerebral palsy) affecting speech output, will be excluded.

Phenomena of interest (Concept)

To be included in the umbrella review, studies must assess children or the outcomes of intervention for children with SSD. This can include articulation disorder, childhood apraxia of speech, (formerly known as developmental verbal dyspraxia) or phonological disorders/delay.

Context

The context for included reviews will be open in that it will consider reviews that retain studies taking place in any setting (e.g., home, clinic, nursery) and geographical location.

Information Sources

For peer review only

As the aim of this umbrella review is to provide a long list of assessments, interventions, outcomes and outcome tools (measures) used in the evaluation of SSD in children, it will not exclude relevant studies on account of their review methodology. However, to maintain a minimum standard of research quality, included reviews will have been published within peer reviewed journals. To locate papers with this minimum quality which have been subject to peer review, grey literature will be excluded. The complete search will include Ovid Medline, OVID Embase, CINAHL, Psychinfo and Cochrane. These databases have been selected because they cover a broad range of journals pertaining to medicine, psychology (including child development) and the allied health professions.

In addition to these standard journal databases other platforms will be integrated including Campbell collaboration, COSMIN, Figshare, JBI, OSF, PROSPERO, Speechbite. Due to a limitation in resources, included studies will be in English. In order to included literature hopefully relevant to current speech and language therapy practice, the search will have a minimum publication year of 2010 (01/01/2010). Where a potentially relevant review article cannot be retrieved, direct contact with the study authors will be made.

Search strategy

In accordance with JBI protocol development guidance an initial limited search of two databases was conducted prior to the full search being carried out.[21] Initially a set of key terms was developed by the first author, in consultation with two independent subject experts with significant postdoctoral research experience in the area. These terms were used for the initial limited search of Ovid Medline and Ovid Embase to identify articles on the topic. With the support of a clinical librarian the text words contained in the articles and abstracts of relevant articles and the index terms used to describe the articles were used to develop a full search strategy for Medline. Table 1 presents the full search strategy for Medline. When completing the database search for the full review, keywords and index terms will be adapted for each selected database as appropriate. The reference list of all included sources of evidence will be screened for additional studies.

Table 1. *Full search strategy for Medline*

1	(child* or youth* or boy* or girl* or juvenil* or teenage* or adolescen* or "young person*" or "young people*" or toddler* or infan* or baby or babies).mp.
2	Child/ or Adolescent/ or Infant/ or Infant, Newborn/
3	1 or 2
4	(phon* or speech or speech disorder* or speech impairment* or speech sound disorder* or speech sound difficult* or speech-sound* or speech retard* or speech delay* or speech disabilit* or speech handicap* or speech problem* or childhood apraxia of speech or apraxia of speech or developmental verbal dyspraxia or verbal dyspraxia or dyspraxia or articulat*).ti,ab.
5	exp Speech Sound Disorder/
6	4 or 5
7	("clinical service*" or "therap* service*" or NHS or "social care" or "social service*" or school* or education* or nurser* or "early year*" or preschool* or pre-school* or college* or universit*).mp.
8	Schools/ or Universities/ or Nurseries, Infant/ or Child, Preschool/ or Social Support/
9	7 or 8
10	(exp META-ANALYSIS AS TOPIC/ or ("meta analy*" or "metaanaly*").ti,ab. or META-ANALYSIS/ or (systematic adj1 (review*1 or overview*1)).ti,ab. or exp REVIEW LITERATURE AS TOPIC/ or (cochrane or embase or psychlit or psyclit or psychinfo or

For peer review only - http://bmjopen.bmj.com/site/about/guidelines.xhtml

	psycinfo or cinahl or cinhal or "science citation index" or bids or cancerlit).ab. or ("reference list*" or bibliograph* or hand-search* or "relevant journals" or "manual search*").ab. or (("selection criter*" or "data extraction").ab. and exp REVIEW/)) not ((ANIMALS/ not (ANIMALS/ and exp HUMANS/)) and (COMMENT/ or LETTER/ or EDITORIAL/ or (letter* or comment*1 or editorial*1).ti,ab.))
11	3 and 6 and 9 and 10
12	11
13	limit 12 to (english language and yr="2010 -Current")

Study/Source of Evidence selection

Following the search, all identified citations will be collated and uploaded into Endnote and duplicates removed. Inclusion and Exclusion criteria are presented in table 2. Titles of studies which are clearly unrelated to the population and concept of the umbrella review will also be removed. Two reviewers will independently review 100% of the remaining abstracts against the inclusion criteria as stated. They will meet to compare their selection of articles. Where disagreement is present the two reviewers will meet to discuss and if consensus is not achieved a third reviewer will be included in the discussion. Once all abstracts have been reviewed, potentially relevant sources for full text review will then be retrieved in full and imported into the Rayyan.ai system for the systematic review management.[24] The two reviewers will examine all selected papers independently at full text level with regular consensus meetings. Reasons for the exclusion of sources at full text will be recorded and reported in the umbrella review. Any disagreements that arise between the reviewers at each stage of the selection process will be resolved through either discussion or with an additional reviewer/s. The results of the search and the study inclusion process will be reported in full in the final umbrella review and presented in a Preferred Reporting Items for Systematic Reviews and Meta-analyses extension for umbrella review flow diagram.[25]

Following final selection and retention of review articles, critical appraisal will be undertaken using the AMSTAR tool.[22] This tool is selected as it is designed to critically appraise systematic reviews that include randomised or non-randomised studies of healthcare interventions, or both. Two reviewers will individually appraise each study, with consensus meetings to confirm ratings. As with the study inclusion process, if consensus cannot be met, a third reviewer will be consulted.

Table 2: Inclusion and Exclusion criteria

**Inclusion Criteria**	**Exclusion Criteria**
• Children of any age<br>• Children with SSD of unknown origin<br>• Children diagnosed with an SSD subtype other than:<br>○ SSD associated with cleft lip and/or palate<br>○ Dysarthria<br>○ acquired SSD	• Reviews not written in English<br>• Reviews that report outcomes for adults<br>• Reviews of studies with no reported assessments or outcomes from interventions for SSD<br>• Reviews reporting speech sound delay.

For peer review only - http://bmjopen.bmj.com/site/about/guidelines.xhtml

○ speech sound delay	

Data Extraction

Data from the retained reviews will be identified using a researcher-developed extraction form. This form was adapted from guidance provided by the JBI Reviewer's Manual in order to meet the specific requirements of the proposed review.[23] The form was piloted by two independent reviewers on 2 relevant studies identified from the initial limited search. A final draft was agreed following a consensus meeting between the two reviewers. The final draft was amended to include specific details about the population and concept as relevant to the aims of this umbrella review. Study design, population details include age, assessment, intervention, comparison, outcome, and context will be extracted. Where these are not presented for each paper in a retained review, the source paper will be obtained, and the data extracted from that. The data extraction tool (Table 3) will be revised if necessary, during the process of extracting data from each included information source.

Table 3. *Extraction form*

**DATA CHARTING**<br>**Evidence source details and characteristics**	
Citation details (reference)	
Study design / type of review	
Country of origin of the review	
Number of articles included in review<br>Primary Research Question<br>Secondary Research Question(s)<br>PICO/PCC Criteria<br>Setting/context<br>Included study designs<br>Inclusion/exclusion	
Demographic items<br>• Age<br>• Biological sex<br>• Diversity characteristics<br>• Setting<br>• Comorbidity<br>• SSD sub-type	
**Interventions**	
Intervention type	

For peer review only - http://bmjopen.bmj.com/site/about/guidelines.xhtml

Intervention method<br>Intervention delivered by<br>Service delivery framework	
Therapeutic Content<br>• Dose<br>• Frequency<br>• Method	
Assessments	
Outcomes	
Measurement instruments	
Analysis performed	
Conclusions drawn	

For peer review only

Analysis of the evidence

The results of each included review paper will not be independently reported as this study is not being conducted within a systematic review methodology.[23] However, as a broad overview of study quality has been included, a brief synthesis of overall study findings will be reported narratively.

The Principles from Grading of Recommendations Assessment, Development, and Evaluation (GRADE) will be used for an overall assessment of the quality of evidence for each intervention or phenomena of interest. The GRADE concept is based on an assessment of the following criteria: quality of primary studies, design of primary studies, consistency and directness.[26]

Presentation of the results

The overall study information with concept and context data will be presented in tabular form with a corresponding narrative summary for each section (tables 4 and 5). The findings from the quality appraisal (AMSTAR) will be discussed narratively, with tables summarising reviewer appraisal ratings. As the presentation of data is an iterative process dependent on study findings,[21] these presentation approaches may be further refined at review stage according to the content of the findings.

Table 4. *Presentation of overarching review information*

Overarching study information and population					
Reference (country)	Type of study	Aims (as relevant to the review)	Number of studies included (PICO/PPC)	No children (biographical information)	Age range at baseline

For peer review only - http://bmjopen.bmj.com/site/about/guidelines.xhtml

Table 5. *Presentation of outcomes and measurement instruments*

Speech Interventions	Speech Assessments	Speech outcomes	Measurement instruments	Analysis performed	Conclusions drawn

Dissemination

This protocol has described the initial limited search process, the development of a usable extraction tool, as well as an overview of how evidence will be analysed and presented. The next stage will be to conduct the full review and report on the findings as in accordance with this protocol.

Authors contributions

First author (SH) devised the search strategy and the data extraction form. They also wrote the first full draft of the manuscript. The second (SB), third (JC), fourth (HS) and fifth (YW) authors, all reviewed, revised and commented on the developed search strategy. All authors reviewed and agreed final manuscript.

Funding statement

The authors of this paper are holders of a Research for Patient Benefit (RfPB) award (NIHR202766) is funded in partnership by the NIHR for this research project. The views expressed in this publication are those of the author(s) and not necessarily those of the NIHR, NHS or the UK Department of Health and Social Care.

Disclosure of competing interests

There are no competing interests for the authors to disclose.

For peer review only

For peer review only - http://bmjopen.bmj.com/site/about/guidelines.xhtml

References

1. American Psychiatric Association, DSM-5 Task Force. Diagnostic and Statistical Manual of Mental Disorders (5th ed.). American Psychiatric Publishing, Inc.2013 https://doi.org/10.1176/appi.books.9780890425596
2. Eadie P, Morgan A, Ukoumunne OC, et al. Speech sound disorder at 4 years: prevalence, comorbidities, and predictors in a community cohort of children. *Dev Med Child Neurol* 2015;57(6):578-584.
3. Shriberg LD, Tomblin JB, McSweeny JL. Prevalence of speech delay in 6-year old children and comorbidity with language impairment. *Journal of Speech, Language & Hearing Research* 1992;42:1461-1481.
4. Wren YE, Miller LL, Peters TJ, et al. Prevalence and predictors of persistent speech sound disorder at eight-years-old: Findings from a population cohort study. *Journal of Speech, Language & Hearing Research* 2016;59:647-673.
5. Broomfield J, Dodd B. Children with speech and language disability: caseload characteristics. *Int J Lang Commun Disord* 2004;39(3):303-24
6. Anthony JL, Aghara RG, Dunkelberger MJ, et al. What factors place children with speech sound disorders at risk for reading problems? *Am J Speech Lang Pathol* 2011;20(2):146-60. doi: 10.1044/1058-0360(2011/10-0053).
7. Beitchman JH, Wilson B, Johnson CJ, et al. Fourteen-Year Follow-up of Speech/Language Impaired and Control Children: Psychiatric Outcome. *Journal of the American Academy of Child & Adolescent Psychiatry* 2001;40(1):75-82.
8. Felsenfeld S, Broen PA, McGue M. A 28-year follow-up of adults with a history of moderate phonological disorder: educational and occupational results. *J Speech Hear Res* 1994;37(6):1341-53. doi: 10.1044/jshr.3706.1341.
9. McCormack J, McLeod S, McAllister L, Harrison LJ. A systematic review of the association between childhood speech impairment and participation across the lifespan. *International Journal of Speech-Language Pathology* 2009;11(2):155-170.
10. Morgan L, Overton S, Bates S, et al. Making the case for the collection of a minimal dataset for children with speech sound disorder. *Int J Lang Commun Disord* 2021;56(5):1097-1107. doi: 10.1111/1460-6984.12649.
11. Morgan, L, Wren YE. A Systematic Review of the Literature on Early Vocalizations and Babbling Patterns in Young Children. *Communication Disorders Quarterly* 2018;40(1), 3–14.
12. RCSLT & ICAN. Bercow: Ten Years On: An independent review of provision for children and young people with speech, language and communication needs in England. 2018. https://www.bercow10yearson.com/wpcontent/uploads/2018/03/337644-ICAN-Bercow-Report-WEB.pdf
13. Roulstone S, Coad J, Ayre A, et al. The Preferred Outcomes of Children with Speech. Language and Communication Needs and their Parents. 2012. Available online at: http://dera. ioe. ac. uk/16329/7/DFE-RR247- BCRP12_Redacted. pdf.
14. Wren YE, Harding S, Goldbart J, Roulstone S. A systematic review and classification of interventions for speech sound disorder in preschool children. International Journal of Language and Communication Disorders 2018;53:446-467.
15. Horwitz RI, Hayes-Conroy A, Caricchio R, Singer BH. From Evidence Based Medicine to Medicine Based Evidence. *The American Journal of Medicine* 2017;130(11):1246-1250.
16. McFaul H, Mulgrew L, Smyth J, Titterington J. Applying evidence to practice by increasing intensity of intervention for children with severe speech sound disorder: a

For peer review only - http://bmjopen.bmj.com/site/about/guidelines.xhtml

quality improvement project. *BMJ Open Quality* 2022;11(2):e001761. https://doi.org/10.1136/BMJOQ-2021-001761
17. Roulstone SE, Marshall JE, Powell GG, et al. Evidence-based intervention for preschool children with primary speech and language impairments: Child Talk – an exploratory mixed-methods study. Southampton (UK): NIHR Journals Library. 2015. PMID: 26312364.
18. Lyons R, Roulstone S. Labels, identity and narratives in children with primary speech and language impairments. *Int J Speech Lang Pathol* 2015;19(5):503-518. doi: 10.1080/17549507.2016.1221455
19. McLeod S, Bleile K. The ICF: a framework for setting goals for children with speech impairment. *Child Language Teaching and Therapy* 2004;20:199-219.
20. Williams C, Harding S, Wren Y. An Exploratory Study of Speech and Language Therapy Intervention for Children Born With Cleft Palate±Lip. *The Cleft Palate-Craniofacial Journal* 2020;p.1055665620954734.
21. Aromataris E, Fernandez R, Godfrey C, et al. Chapter 10: Umbrella Reviews. In: Aromataris E, Munn Z (Editors). JBI Manual for Evidence Synthesis. *JBI* 2020;Available from https://synthesismanual.jbi.global. https://doi.org/10.46658/JBIMES-20-11
22. Shea BJ, Reeves BC, Wells G, et al. AMSTAR 2: a critical appraisal tool for systematic reviews that include randomised or non-randomised studies of healthcare interventions, or both. *BMJ* 2017;358 :j4008 doi:10.1136/bmj.j4008
23. Aromataris E, Munn Z. Chapter 1: JBI Systematic Reviews. In: Aromataris E, Munn Z (Editors). JBI Manual for Evidence Synthesis. *JBI* 2020, Available from https://synthesismanual.jbi.global. https://doi.org/10.46658/JBIMES-20-02
24. Ouzzani M, Hammady H, Fedorowicz Z, Elmagarmid A. Rayyan — a web and mobile app for systematic reviews. *Systematic Reviews* 2016;5:210, doi: 10.1186/s13643-016-0384-4.
25. Page MJ, McKenzie JE, Bossuyt PM, et al. The PRISMA 2020 statement: an updated guideline for reporting systematic reviews. *BMJ* 2021;372:n71. doi: 10.1136/bmj.n71.
26. Guyatt GH, Oxman AD, Vist GE, et al. GRADE: an emerging consensus on rating quality of evidence and strength of recommendations. *BMJ* 2008;336(7650):924-6. doi: 10.1136/bmj.39489.470347.AD.

For peer review only - http://bmjopen.bmj.com/site/about/guidelines.xhtml

PRISMA 2020 Checklist

Section and Topic	Item #	Checklist item	Location where item is reported
**TITLE**			
Title	1	Identify the report as a systematic review.	Title
**ABSTRACT**			
Abstract	2	See the PRISMA 2020 for Abstracts checklist.	-
**INTRODUCTION**			
Rationale	3	Describe the rationale for the review in the context of existing knowledge.	Page 3
Objectives	4	Provide an explicit statement of the objective(s) or question(s) the review addresses.	Page 4
**METHODS**			
Eligibility criteria	5	Specify the inclusion and exclusion criteria for the review and how studies were grouped for the syntheses.	Page 4
Information sources	6	Specify all databases, registers, websites, organisations, reference lists and other sources searched or consulted to identify studies. Specify the date when each source was last searched or consulted.	Page 4 & 5
Search strategy	7	Present the full search strategies for all databases, registers and websites, including any filters and limits used.	Page 5
Selection process	8	Specify the methods used to decide whether a study met the inclusion criteria of the review, including how many reviewers screened each record and each report retrieved, whether they worked independently, and if applicable, details of automation tools used in the process.	Page 6
Data collection process	9	Specify the methods used to collect data from reports, including how many reviewers collected data from each report, whether they worked independently, any processes for obtaining or confirming data from study investigators, and if applicable, details of automation tools used in the process.	Page 6
Data items	10a	List and define all outcomes for which data were sought. Specify whether all results that were compatible with each outcome domain in each study were sought (e.g. for all measures, time points, analyses), and if not, the methods used to decide which results to collect.	Table 3
	10b	List and define all other variables for which data were sought (e.g. participant and intervention characteristics, funding sources). Describe any assumptions made about any missing or unclear information.	Table 3
Study risk of bias assessment	11	Specify the methods used to assess risk of bias in the included studies, including details of the tool(s) used, how many reviewers assessed each study and whether they worked independently, and if applicable, details of automation tools used in the process.	Page 8
Effect measures	12	Specify for each outcome the effect measure(s) (e.g. risk ratio, mean difference) used in the synthesis or presentation of results.	-
Synthesis methods	13a	Describe the processes used to decide which studies were eligible for each synthesis (e.g. tabulating the study intervention characteristics and comparing against the planned groups for each synthesis (item #5)).	Page 8
	13b	Describe any methods required to prepare the data for presentation or synthesis, such as handling of missing summary statistics, or data conversions.	Page 8
	13c	Describe any methods used to tabulate or visually display results of individual studies and syntheses.	Page 8
	13d	Describe any methods used to synthesize results and provide a rationale for the choice(s). If meta-analysis was performed, describe the model(s), method(s) to identify the presence and extent of statistical heterogeneity, and software package(s) used.	Page 8
	13e	Describe any methods used to explore possible causes of heterogeneity among study results (e.g. subgroup analysis, meta-regression).	Page 8
	13f	Describe any sensitivity analyses conducted to assess robustness of the synthesized results.	Page 8
Reporting bias assessment	14	Describe any methods used to assess risk of bias due to missing results in a synthesis (arising from reporting biases).	Page 8
Certainty assessment	15	Describe any methods used to assess certainty (or confidence) in the body of evidence for an outcome.	Page 8

For peer review only - http://bmjopen.bmj.com/site/about/guidelines.xhtml

PRISMA 2020 Checklist

Section and Topic	Item #	Checklist item	Location where item is reported
**RESULTS**			
Study selection	16a	Describe the results of the search and selection process, from the number of records identified in the search to the number of studies included in the review, ideally using a flow diagram.	
	16b	Cite studies that might appear to meet the inclusion criteria, but which were excluded, and explain why they were excluded.	
Study characteristics	17	Cite each included study and present its characteristics.	
Risk of bias in studies	18	Present assessments of risk of bias for each included study.	
Results of individual studies	19	For all outcomes, present, for each study: (a) summary statistics for each group (where appropriate) and (b) an effect estimate and its precision (e.g. confidence/credible interval), ideally using structured tables or plots.	
Results of syntheses	20a	For each synthesis, briefly summarise the characteristics and risk of bias among contributing studies.	
	20b	Present results of all statistical syntheses conducted. If meta-analysis was done, present for each the summary estimate and its precision (e.g. confidence/credible interval) and measures of statistical heterogeneity. If comparing groups, describe the direction of the effect.	
	20c	Present results of all investigations of possible causes of heterogeneity among study results.	
	20d	Present results of all sensitivity analyses conducted to assess the robustness of the synthesized results.	
Reporting biases	21	Present assessments of risk of bias due to missing results (arising from reporting biases) for each synthesis assessed.	
Certainty of evidence	22	Present assessments of certainty (or confidence) in the body of evidence for each outcome assessed.	
**DISCUSSION**			
Discussion	23a	Provide a general interpretation of the results in the context of other evidence.	
	23b	Discuss any limitations of the evidence included in the review.	
	23c	Discuss any limitations of the review processes used.	
	23d	Discuss implications of the results for practice, policy, and future research.	
**OTHER INFORMATION**			
Registration and protocol	24a	Provide registration information for the review, including register name and registration number, or state that the review was not registered.	Abstract & Page 5
	24b	Indicate where the review protocol can be accessed, or state that a protocol was not prepared.	Abstract & Page 5
	24c	Describe and explain any amendments to information provided at registration or in the protocol.	
Support	25	Describe sources of financial or non-financial support for the review, and the role of the funders or sponsors in the review.	Page 9
Competing interests	26	Declare any competing interests of review authors.	Page 9
Availability of data, code and other materials	27	Report which of the following are publicly available and where they can be found: template data collection forms; data extracted from included studies; data used for all analyses; analytic code; any other materials used in the review.	

For peer review only

PRISMA 2020 Checklist

From: Page MJ, McKenzie JE, Bossuyt PM, Boutron I, Hoffmann TC, Mulrow CD, et al. The PRISMA 2020 statement: an updated guideline for reporting systematic reviews. BMJ 2021;372:n71. doi: 10.1136/bmj.n71

For more information, visit: http://www.prisma-statement.org/

For peer review only

BMJ Open

Outcome measures for Children with Speech Sound Disorder: An Umbrella Review Protocol

Journal:	*BMJ Open*
Manuscript ID	bmjopen-2022-068945.R1
Article Type:	Protocol
Date Submitted by the Author:	04-Jan-2023
Complete List of Authors:	Harding, Sam; North Bristol NHS Trust, Bristol Speech and Language Therapy Research Unit; North Bristol NHS Trust, Research and Innoviation<br>Burr, Sam; North Bristol NHS Trust, Bristol Speech and Language Therapy Research Unit<br>Cleland, Joanne; University of Strathclyde, Psychological Sciences and Health<br>Stringer, Helen; Newcastle University<br>Wren, Yvonne; North Bristol NHS Trust, Bristol Speech & Language Therapy Research Unit; University of Bristol, Bristol Dental School
**Primary Subject Heading**:	Evidence based practice
Secondary Subject Heading:	Paediatrics, Communication
Keywords:	Protocols & guidelines < HEALTH SERVICES ADMINISTRATION & MANAGEMENT, Speech pathology < OTOLARYNGOLOGY, Health policy < HEALTH SERVICES ADMINISTRATION & MANAGEMENT

SCHOLARONE™
Manuscripts

Outcome Measures for Children with Speech Sound Disorder: An Umbrella Review Protocol

Harding, S.A.[1,2], Burr, S. [1,2], Cleland, J.[3], Stringer, H.[4], Wren, Y.[1,5,6]

Corresponding Author:
Dr Sam Harding, Research Fellow
[1]Bristol Speech and Language Therapy Research Unit, North Bristol NHS Trust, Southmead Hospital, Bristol, BS10 5NB
[2]Research and Innovation North Bristol NHS Trust, Southmead Hospital, Bristol, BS10 5NB
Email: sharding.jb@gmail.com
ORCiD: 0000-0002-5870-2094

Authors:
Dr Sam Burr
[1]Bristol Speech and Language Therapy Research Unit, North Bristol NHS Trust, Southmead Hospital, Bristol, BS10 5NB
[2]North Bristol NHS Trust, Southmead Hospital, Bristol, BS10 5NB
Email: sam.burr@nbt.nhs.uk
ORCiD: 0000-0002-4177-2459

Dr Joanne Cleland
[3]Psychological Sciences and Health, Graham Hills Building, University of Strathclyde, Glasgow, Scotland
Email: joanne.cleland@strath.ac.uk
ORCiD: 0000-0002-0660-1646

Dr Helen Stringer
[4]King George VI Building, Newcastle University, Queen Victoria Road, Newcastle upon Tyne, NE1 7RU
Email: helen.stringer@ncl.ac.uk
ORCiD: 0000-0002-7470-2166

Dr Yvonne Wren
[1]Bristol Speech and Language Therapy Research Unit, North Bristol NHS Trust, Southmead Hospital, Bristol, BS10 5NB
[5] Cardiff School of Sport and Health Sciences, Cardiff Metropolitan University, Llandaff Campus, Western Avenue, Cardiff, Wales CF5 2YB
[6] Bristol Dental School, University of Bristol, Lower Maudlin St, Bristol, UK, BS1 2LY Email:
Email: Yvonne.Wren@Bristol.ac.uk
ORCiD: 0000-0002-1575-453X

Word Count: 2281

Key words: Umbrella review, protocol, speech sound disorder, assessment, intervention, outcomes

For peer review only

Outcome Measures for Children with Speech Sound Disorder: An Umbrella Review Protocol

Abstract

Introduction: Speech sound disorder (SSD) describes a 'persistent difficulty with speech sound production that interferes with speech intelligibility or prevents verbal communication'. There is a need to establish which care pathways are most effective and efficient for children with SSD. Comparison of care pathways requires clearly defined, evidence-based, interventions and agreement on how to measure the outcomes. At present no list of assessments, interventions or outcomes exists.

The objective of this paper is to provide a rigorous and detailed protocol for an umbrella review of assessments, interventions, and outcomes which target SSD in children. The protocol details the development of a search strategy, and trial of an extraction tool.

Methods and Analyses: The umbrella review has been registered with PROSPERO (CRD42022316284). Papers included can use a review methodology of any sort, but must include children of any age, with a SSD of unknown origin. In accordance with the Joanna Briggs institute scoping review methods guidelines, an initial search of the Ovid Emcare and Ovid Medline databases was conducted. Following this a final search strategy for these databases were produced. A draft extraction form was developed.

Ethics and Dissemination: Ethical approval is not needed for an umbrella review protocol. Following the systematic development of an initial search strategy and extraction form, an umbrella review of this topic can take place. Dissemination of findings will be through peer-reviewed publications, social media, and patient and public engagement.

Strengths and limitations of this study

- **Strength:** The number of systematic reviews produced to inform healthcare has rapidly increased in recent years, leading to healthcare decision makers needing to source multiple articles. The proposed umbrella review is designed to collate existing systematic reviews.
- **Strength:** This protocol follows the Preferred Reporting Items for Systematic Review and Meta-Analysis Protocols (PRISMA-P) guidelines.
- **Strength:** This protocol is also guided by the COS-STAP checklist due to its aim of defining a core outcome set.
- **Limitation:** Electronic databases in languages other than English will not be searched. This may cause language bias.

For peer review only - http://bmjopen.bmj.com/site/about/guidelines.xhtml

Introduction

Terminology and prevalence

Speech sound disorder (SSD) describes a 'persistent difficulty with speech sound production that interferes with speech intelligibility or prevents verbal communication'.[1] Prevalence is high, with estimates of 3.6% in children aged 4-8,[2-4] and upwards of 76,000 children referred to National Health Service (NHS) Speech and Language Therapy (SLT) services annually.[5] While a minority of children with SSD have clear aetiology (e.g., cleft palate, cerebral palsy, hearing impairment), in most cases, SSD has no identifiable cause and the evidence for intervention for this group is limited. Untreated, the impact of SSD is far reaching, leading to poor outcomes in education, employment and mental health.[6-9] NHS SLT is provided to children with SSD via a range of care pathways, typically defined by resource constraints, rather than robust evidence. Vanhaecht *et al* defined a care pathway as "a complex intervention for the mutual decision-making and organisation of care processes for a well-defined group of patients during a well-defined period." [10] Care pathways aim to improve care, outcomes and patient satisfaction while also optimising the use of resources.[10] Carepathway examples include total hip replacement and palliative care.

Implications for clinical practice

There is a need to establish which care pathways are most effective and efficient for children with SSD. Comparison of care pathways requires both clearly defined, evidence-based, interventions and agreement on how best to measure the outcomes of these interventions for children with SSD. However, a review of existing case notes of children treated for SSD were found to be too incomplete to compare pathways.[11] Additionally, it was found that pre- and post-intervention data and variables recorded in the clinical case notes varied significantly between and within SLT services, thereby negating comparison. As a first step to determining which care pathways are most effective and efficient, this umbrella review will ask which assessment and outcome measures are commonly employed with children with SSD. Moreover, Morgan et al., suggests that there is a need for agreement on a national core outcome set (COS) for SSD.[12] A national consultation in 2018 identified the need to collect consistent data and recommended that NHS England support providers to collect data on the quality and outcomes of interventions (recommendation 4.5, p.29).[13]

Reviews to date

Evidence from systematic reviews and trials has shown that intervention is effective for the majority of children with SSD and that these children do not make progress without intervention.[14,15] However, studies have typically employed intervention protocols which are intense and are difficult to replicate in NHS SLT services.[16-1814] Moreover, unlike research studies, clinical intervention takes place within care pathways which vary in terms of timing of intervention (pre-school, school age), delivery by speech and language therapists (SLTs) or assistants, number, frequency and duration of sessions, and involvement of parents or education staff, as well as the assessments and outcome measures used.

Previous research has identified that functional goals such as independence and improved social interaction are of greatest importance to parents,[18] while children listed improved

For peer review only - http://bmjopen.bmj.com/site/about/guidelines.xhtml

speech alongside improved behaviour, schoolwork and skill at sports as well as making friends as important goals.[19, 20] Preferred outcomes for pre-school children with SSD amongst SLTs have been identified as: intelligibility, social interaction and participation.[21] However, although a list of assessment tools (i.e., the tools used to obtain a speech sample) was compiled as part of this work,[21] it did not consider which specific analysis (e.g., percentage consonants correct, consonant inventory, error pattern analysis) is preferred to measure the primary outcome or how this relates to functional domains (e.g., social interaction, participation, inclusion).

As can be seen from the number of reviews included in this introduction, evidence syntheses undertaken using this type of methodology are increasing in frequency in published literature. They provide a rigorous and transparent knowledge base for translating clinical research into decisions, and as such are 'go to' documents to advise healthcare service construction and evaluation. An overarching review which combines previous reviews is needed for clinicians and researchers to consolidate what we know about interventions in SSD to date. Umbrella reviews are reviews of previously published scoping reviews, systematic reviews or meta-analyses. They aim to collate and represent one of the highest levels of evidence synthesis currently available, undertaking at least in part the historical role of the systematic review.[22,23] It is in recognition of the number of potential reviews previously undertaken in the field of childhood SSD that the proposed review being outlined in the current protocol used an Umbrella methodology.

Review objective

The objective of the proposed umbrella review is to collate the tools used for initial and baseline assessment, intervention and outcome measurement with children with SSD in speech and language therapy.

Review question

What assessment, interventions and outcomes are reported for children with SSD in health, social care and education?

Methods and Analysis

The review will be conducted in accordance with the Joanna Briggs Institute (JBI) methodology for umbrella reviews, [24] with an addition relating to quality appraisal where the AMSTAR (Assessment of Multiple Systematic Reviews) tool will be used. [25] This is a literature review and therefore ethical approval is not required. The umbrella review has been registered with PROSPERO (CRD42022316284).

Patient and Public Involvement

None.

Eligibility criteria

In line with the JBI guidance, the eligibility for included studies will be outlined according to population, phenomena of interest and context of data.[24] As this will be an umbrella review, the only papers retained for inclusion will be reviews. This can include any type of review, e.g., systematic reviews of effectiveness, mixed methods, qualitative, scoping reviews.[26]

Population

For peer review only - http://bmjopen.bmj.com/site/about/guidelines.xhtml

The included population is children of any age (from birth up to 18 years) with a diagnosis of SSD. Studies will not be excluded if the interventions include additional intervention targets (e.g., for receptive language). Children whose speech needs are associated with a biomedical condition with a known association with communication, such as sensorineural deafness, autistic spectrum condition or cleft lip and palate and neurological conditions (e.g., cerebral palsy) affecting speech output, will be excluded.

Phenomena of interest (Concept)

To be included in the umbrella review, studies must assess children or the outcomes of intervention for children with SSD. This can include articulation disorder, childhood apraxia of speech, (formerly known as developmental verbal dyspraxia) or phonological disorders/delay. It will exclude children with a known cause for their SSD, such as those with identified genetic or chromosomal anomalies, and congenital or acquired neurological conditions.

Context

The context for included reviews will be open in that it will consider reviews that retain studies taking place in any setting (e.g., home, clinic, nursery) and geographical location.

Information Sources

As the aim of this umbrella review is to provide a long list of assessments, interventions, outcomes and outcome tools (measures) used in the evaluation of SSD in children, it will not exclude relevant studies on account of their review methodology. However, to maintain a minimum standard of research quality, included reviews will have been published within peer reviewed journals. To locate papers with this minimum quality which have been subject to peer review, grey literature will be excluded. The complete search will include Ovid Medline, Ovid Embase, CINAHL (Cumulative Index to Nursing and Allied Health Literature), Psychinfo and Cochrane. These databases have been selected because they cover a broad range of journals pertaining to medicine, psychology (including child development) and the allied health professions.

In addition to these standard journal databases other platforms will be integrated including Campbell collaboration, COSMIN (COnsensus-based Standards for the selection of health Measurement INstruments), Figshare, JBI, OSF (Open Science Framework), PROSPERO, Speechbite. Due to a limitation in resources, included studies will be in English. In order to included literature hopefully relevant to current speech and language therapy practice, the search will have a minimum publication year of 2010 (01/01/2010). Where a potentially relevant review article cannot be retrieved, direct contact with the study authors will be made.

Search strategy

In accordance with JBI protocol development guidance an initial limited search of two databases was conducted prior to the full search being carried out.[24] Initially a set of key terms was developed by the first author, in consultation with two independent subject experts with significant postdoctoral research experience in the area. These terms were used for the initial limited search of Ovid Medline and Ovid Embase to identify articles on the topic. With the support of a clinical librarian the text words contained in the articles and abstracts of relevant articles and the index terms used to describe the articles were

For peer review only - http://bmjopen.bmj.com/site/about/guidelines.xhtml

used to develop a full search strategy for Medline. Table 1 presents the full search strategy for Medline. When completing the database search for the full review, keywords and index terms will be adapted for each selected database as appropriate. The reference list of all included sources of evidence will be screened for additional studies.

Table 1. *Full search strategy for Medline*

1	(child* or youth* or boy* or girl* or juvenil* or teenage* or adolescen* or "young person*" or "young people*" or toddler* or infan* or baby or babies).mp.
2	Child/ or Adolescent/ or Infant/ or Infant, Newborn/
3	1 or 2
4	(phon* or speech or speech disorder* or speech impairment* or speech sound disorder* or speech sound difficult* or speech-sound* or speech retard* or speech delay* or speech disabilit* or speech handicap* or speech problem* or childhood apraxia of speech or apraxia of speech or developmental verbal dyspraxia or verbal dyspraxia or dyspraxia or articulat*).ti,ab.
5	exp Speech Sound Disorder/
6	4 or 5
7	("clinical service*" or "therap* service*" or NHS or "social care" or "social service*" or school* or education* or nurser* or "early year*" or preschool* or pre-school* or college* or universit*).mp.
8	Schools/ or Universities/ or Nurseries, Infant/ or Child, Preschool/ or Social Support/
9	7 or 8
10	(exp META-ANALYSIS AS TOPIC/ or ("meta analy*" or "metaanaly*").ti,ab. or META-ANALYSIS/ or (systematic adj1 (review*1 or overview*1)).ti,ab. or exp REVIEW LITERATURE AS TOPIC/ or (cochrane or embase or psychlit or psyclit or psychinfo or psycinfo or cinahl or cinhal or "science citation index" or bids or cancerlit).ab. or ("reference list*" or bibliograph* or hand-search* or "relevant journals" or "manual search*").ab. or (("selection criter*" or "data extraction").ab. and exp REVIEW/)) not ((ANIMALS/ not (ANIMALS/ and exp HUMANS/)) and (COMMENT/ or LETTER/ or EDITORIAL/ or (letter* or comment*1 or editorial*1).ti,ab.))
11	3 and 6 and 9 and 10
12	11
13	limit 12 to (english language and yr="2010 -Current")

Study/Source of Evidence selection

Following the search, all identified citations will be collated and uploaded into Endnote and duplicates removed. Inclusion and Exclusion criteria are presented in table 2. Titles of studies which are clearly unrelated to the population and concept of the umbrella review will also be removed. Two reviewers will independently review 100% of the remaining abstracts against the inclusion criteria as stated. They will meet to compare their selection of articles. Where disagreement is present the two reviewers will meet to discuss and if consensus is not achieved a third reviewer will be included in the discussion. Once all abstracts have been reviewed, potentially relevant sources for full text review will then be retrieved in full and imported into the Rayyan.ai system for the systematic review management. [27] The two reviewers will examine all selected papers independently at full text level with regular consensus meetings. Reasons for the exclusion of sources at full text will be recorded and reported in the umbrella review. Any disagreements that arise between the reviewers at each stage of the selection process will be resolved through either discussion or with an additional reviewer/s. The results of the search and the study

For peer review only - http://bmjopen.bmj.com/site/about/guidelines.xhtml

inclusion process will be reported in full in the final umbrella review and presented in a Preferred Reporting Items for Systematic Reviews and Meta-analyses extension for umbrella review flow diagram. [28]

Following final selection and retention of review articles, critical appraisal will be undertaken using the AMSTAR tool. [25] This tool is selected as it is designed to critically appraise systematic reviews that include randomised or non-randomised studies of healthcare interventions, or both. Two reviewers will individually appraise each study, with consensus meetings to confirm ratings. As with the study inclusion process, if consensus cannot be met, a third reviewer will be consulted.

Table 2: Inclusion and Exclusion criteria

**Inclusion Criteria**	**Exclusion Criteria**
• Children of any age<br>• Children with SSD of unknown origin including:<br>• Childhood Apraxia of Speech/ Developmental Verbal Dyspraxia<br>• Articulation disorders<br>• Phonological disorders of all types	• Children with SSDs associated with a biomedical condition for example:<br>• SSD associated with cleft lip and/or palate<br>• Cerebral Palsy<br>• Traumatic brain injury<br>• Reviews not written in English<br>• Reviews that report outcomes for adults<br>• Reviews of studies with no reported assessments or outcomes from interventions for SSD

Data Extraction

Data from the retained reviews will be identified using a researcher-developed extraction form. This form was adapted from guidance provided by the JBI Reviewer's Manual in order to meet the specific requirements of the proposed review. [26] The form was piloted by two independent reviewers on 2 relevant studies identified from the initial limited search. A final draft was agreed following a consensus meeting between the two reviewers. The final draft was amended to include specific details about the population and concept as relevant to the aims of this umbrella review. Study design, population details include age, assessment, intervention, comparison, outcome, and context will be extracted. Where these are not presented for each paper in a retained review, the source paper will be obtained, and the data extracted from that. The data extraction tool (Table 3) will be revised if necessary, during the process of extracting data from each included information source.

Table 3. *Extraction form*

**DATA CHARTING**<br>**Evidence source details and characteristics**	
Citation details (reference)	

For peer review only - http://bmjopen.bmj.com/site/about/guidelines.xhtml

Study design / type of review	
Country of origin of the review	
Number of articles included in review<br>Primary Research Question<br>Secondary Research Question(s)<br>PICO/PCC Criteria<br>Setting/context<br>Included study designs<br>Inclusion/exclusion	
Demographic items<br>• Age<br>• Biological sex<br>• Diversity characteristics<br>• Setting<br>• Comorbidity<br>• SSD sub-type	
**Interventions**	
Intervention type<br>Intervention method<br>Intervention delivered by<br>Service delivery framework<br>Service delivery format(s)	
Therapeutic Content / Therapeutic Dosage<br>• Dose<br>• Frequency<br>• Method	
Assessments	
Outcomes	
Measurement instruments	
Analysis performed	
Conclusions drawn	

Analysis of the evidence

The results of each included review paper will not be independently reported as this study is not being conducted within a systematic review methodology. [26] However, as a broad

For peer review only - http://bmjopen.bmj.com/site/about/guidelines.xhtml

overview of study quality has been included, a brief synthesis of overall study findings will be reported narratively.

The Principles from Grading of Recommendations Assessment, Development, and Evaluation (GRADE) will be used for an overall assessment of the quality of evidence for each intervention or phenomena of interest. The GRADE concept is based on an assessment of the following criteria: quality of primary studies, design of primary studies, consistency and directness. [29]

Presentation of the results

The overall study information with concept and context data will be presented in tabular form with a corresponding narrative summary for each section (tables 4 and 5). The findings from the quality appraisal (AMSTAR) will be discussed narratively, with tables summarising reviewer appraisal ratings. As the presentation of data is an iterative process dependent on study findings, [24] these presentation approaches may be further refined at review stage according to the content of the findings.

Table 4. *Presentation of overarching review information*

Overarching study information and population					
Reference (country)	Type of study	Aims (as relevant to the review)	Number of studies included (PICO/PPC)	No children (biographical information)	Age range at baseline

Table 5. *Presentation of outcomes and measurement instruments*

Speech Interventions	Speech Assessments	Speech outcomes	Measurement instruments	Analysis performed	Conclusions drawn

Dissemination

This protocol has described the initial limited search process, the development of a usable extraction tool, as well as an overview of how evidence will be analysed and presented. The next stage will be to conduct the full review and report on the findings as in accordance with this protocol.

Authors contributions

For peer review only - http://bmjopen.bmj.com/site/about/guidelines.xhtml

First author (SH) devised the search strategy and the data extraction form. They also wrote the first full draft of the manuscript. The second (SB), third (JC), fourth (HS) and fifth (YW) authors, all reviewed, revised and commented on the developed search strategy. All authors reviewed and agreed final manuscript.

Funding statement

The authors of this paper are holders of a Research for Patient Benefit (RfPB) award (NIHR202766) is funded in partnership by the NIHR for this research project. The views expressed in this publication are those of the author(s) and not necessarily those of the NIHR, NHS or the UK Department of Health and Social Care.

Disclosure of competing interests

There are no competing interests for the authors to disclose.

For peer review only

References

1. American Psychiatric Association, DSM-5 Task Force. Diagnostic and Statistical Manual of Mental Disorders (5th ed.). American Psychiatric Publishing, Inc.2013 https://doi.org/10.1176/appi.books.9780890425596
2. Eadie P, Morgan A, Ukoumunne OC, et al. Speech sound disorder at 4 years: prevalence, comorbidities, and predictors in a community cohort of children. *Dev Med Child Neurol* 2015;57(6):578-584.
3. Shriberg LD, Tomblin JB, McSweeny JL. Prevalence of speech delay in 6-year old children and comorbidity with language impairment. *Journal of Speech, Language & Hearing Research* 1992;42:1461-1481.
4. Wren YE, Miller LL, Peters TJ, et al. Prevalence and predictors of persistent speech sound disorder at eight-years-old: Findings from a population cohort study. *Journal of Speech, Language & Hearing Research* 2016;59:647-673.
5. Broomfield J, Dodd B. Children with speech and language disability: caseload characteristics. *Int J Lang Commun Disord* 2004;39(3):303-24
6. Anthony JL, Aghara RG, Dunkelberger MJ, et al. What factors place children with speech sound disorders at risk for reading problems? *Am J Speech Lang Pathol* 2011;20(2):146-60. doi: 10.1044/1058-0360(2011/10-0053).
7. Beitchman JH, Wilson B, Johnson CJ, et al. Fourteen-Year Follow-up of Speech/Language Impaired and Control Children: Psychiatric Outcome. *Journal of the American Academy of Child & Adolescent Psychiatry* 2001;40(1):75-82.
8. Felsenfeld S, Broen PA, McGue M. A 28-year follow-up of adults with a history of moderate phonological disorder: educational and occupational results. *J Speech Hear Res* 1994;37(6):1341-53. doi: 10.1044/jshr.3706.1341.
9. McCormack J, McLeod S, McAllister L, Harrison LJ. A systematic review of the association between childhood speech impairment and participation across the lifespan. *International Journal of Speech-Language Pathology* 2009;11(2):155-170.
10. Vanhaecht K, De Witte K, Sermeus W. The impact of clinical pathways on the organisation of care processes. PhD dissertation, Belgium: KU Leuven; 2007.
11. Morgan L, Overton S, Bates S, et al. Making the case for the collection of a minimal dataset for children with speech sound disorder. *Int J Lang Commun Disord* 2021;56(5):1097-1107. doi: 10.1111/1460-6984.12649.
12. Morgan, L, Wren YE. A Systematic Review of the Literature on Early Vocalizations and Babbling Patterns in Young Children. *Communication Disorders Quarterly* 2018;40(1), 3–14.
13. RCSLT & ICAN. Bercow: Ten Years On: An independent review of provision for children and young people with speech, language and communication needs in England. 2018. https://www.bercow10yearson.com/wpcontent/uploads/2018/03/337644-ICAN-Bercow-Report-WEB.pdf
14. Roulstone S, Coad J, Ayre A, et al. The Preferred Outcomes of Children with Speech. Language and Communication Needs and their Parents. 2012. Available online at: http://dera. ioe. ac. uk/16329/7/DFE-RR247- BCRP12_Redacted. pdf.
15. Wren YE, Harding S, Goldbart J, Roulstone S. A systematic review and classification of interventions for speech sound disorder in preschool children. International Journal of Language and Communication Disorders 2018;53:446-467.
16. Horwitz RI, Hayes-Conroy A, Caricchio R, Singer BH. From Evidence Based Medicine to Medicine Based Evidence. *The American Journal of Medicine* 2017;130(11):1246-1250.

For peer review only - http://bmjopen.bmj.com/site/about/guidelines.xhtml

17. McFaul H, Mulgrew L, Smyth J, Titterington J. Applying evidence to practice by increasing intensity of intervention for children with severe speech sound disorder: a quality improvement project. *BMJ Open Quality* 2022;11(2):e001761. https://doi.org/10.1136/BMJOQ-2021-001761
18. Roulstone SE, Marshall JE, Powell GG, et al. Evidence-based intervention for preschool children with primary speech and language impairments: Child Talk – an exploratory mixed-methods study. Southampton (UK): NIHR Journals Library. 2015. PMID: 26312364.
19. Lyons R, Roulstone S. Labels, identity and narratives in children with primary speech and language impairments. *Int J Speech Lang Pathol* 2015;19(5):503-518. doi: 10.1080/17549507.2016.1221455
20. McLeod S, Bleile K. The ICF: a framework for setting goals for children with speech impairment. *Child Language Teaching and Therapy* 2004;20:199-219.
21. Williams C, Harding S, Wren Y. An Exploratory Study of Speech and Language Therapy Intervention for Children Born With Cleft Palate±Lip. *The Cleft Palate-Craniofacial Journal* 2020;p.1055665620954734.
22. Choi GJ, Kang H. The umbrella review: a useful strategy in the rain of evidence. Korean J Pain. 2022 Apr 1;35(2):127-128. doi: 10.3344/kjp.2022.35.2.127. PMID: 35354675; PMCID: PMC8977198
23. Fusar-Poli P, Radua J. Ten simple rules for conducting umbrella reviews Evidence-Based Mental Health 2018;21:95-100.
24. Aromataris E, Fernandez R, Godfrey C, et al. Chapter 10: Umbrella Reviews. In: Aromataris E, Munn Z (Editors). JBI Manual for Evidence Synthesis. *JBI* 2020;Available from https://synthesismanual.jbi.global. https://doi.org/10.46658/JBIMES-20-11
25. Shea BJ, Reeves BC, Wells G, et al. AMSTAR 2: a critical appraisal tool for systematic reviews that include randomised or non-randomised studies of healthcare interventions, or both. *BMJ* 2017;358 :j4008 doi:10.1136/bmj.j4008
26. Aromataris E, Munn Z. Chapter 1: JBI Systematic Reviews. In: Aromataris E, Munn Z (Editors). JBI Manual for Evidence Synthesis. *JBI* 2020, Available from https://synthesismanual.jbi.global. https://doi.org/10.46658/JBIMES-20-02
27. Ouzzani M, Hammady H, Fedorowicz Z, Elmagarmid A. Rayyan — a web and mobile app for systematic reviews. *Systematic Reviews* 2016;5:210, doi: 10.1186/s13643-016-0384-4.
28. Page MJ, McKenzie JE, Bossuyt PM, et al. The PRISMA 2020 statement: an updated guideline for reporting systematic reviews. *BMJ* 2021;372:n71. doi: 10.1136/bmj.n71.
29. Guyatt GH, Oxman AD, Vist GE, et al. GRADE: an emerging consensus on rating quality of evidence and strength of recommendations. *BMJ* 2008;336(7650):924-6. doi: 10.1136/bmj.39489.470347.AD.

For peer review only

PRISMA-P (Preferred Reporting Items for Systematic review and Meta-Analysis Protocols) 2015 checklist: recommended items to address in a systematic review protocol*

Section and topic	Item No	Checklist item	
**ADMINISTRATIVE INFORMATION**			
Title:			
Identification	1a	Identify the report as a protocol of a systematic review	Identified as an umbrella review (page 1 & 2)
Update	1b	If the protocol is for an update of a previous systematic review, identify as such	N/A
Registration	2	If registered, provide the name of the registry (such as PROSPERO) and registration number	Page 4
Authors:			
Contact	3a	Provide name, institutional affiliation, e-mail address of all protocol authors; provide physical mailing address of corresponding author	Page 1
Contributions	3b	Describe contributions of protocol authors and identify the guarantor of the review	Page 10
Amendments	4	If the protocol represents an amendment of a previously completed or published protocol, identify as such and list changes; otherwise, state plan for documenting important protocol amendments	N/A
Support:			
Sources	5a	Indicate sources of financial or other support for the review	Page 10
Sponsor	5b	Provide name for the review funder and/or sponsor	Page 10
Role of sponsor or funder	5c	Describe roles of funder(s), sponsor(s), and/or institution(s), if any, in developing the protocol	N/A
**INTRODUCTION**			
Rationale	6	Describe the rationale for the review in the context of what is already known	Page 3, reviews to date
Objectives	7	Provide an explicit statement of the question(s) the review will address with reference to participants, interventions, comparators, and outcomes (PICO)	Page 4, Objectives
**METHODS**			
Eligibility criteria	8	Specify the study characteristics (such as PICO, study design, setting, time frame) and report characteristics (such as years considered, language, publication status) to be used as criteria for eligibility for the review	Page 5, PCC

For peer review only

Information sources	9	Describe all intended information sources (such as electronic databases, contact with study authors, trial registers or other grey literature sources) with planned dates of coverage	Page 5
Search strategy	10	Present draft of search strategy to be used for at least one electronic database, including planned limits, such that it could be repeated	Page 5 & 6
Study records:			
Data management	11a	Describe the mechanism(s) that will be used to manage records and data throughout the review	Page 6
Selection process	11b	State the process that will be used for selecting studies (such as two independent reviewers) through each phase of the review (that is, screening, eligibility and inclusion in meta-analysis)	Page 6, table 2
Data collection process	11c	Describe planned method of extracting data from reports (such as piloting forms, done independently, in duplicate), any processes for obtaining and confirming data from investigators	Page 7
Data items	12	List and define all variables for which data will be sought (such as PICO items, funding sources), any pre-planned data assumptions and simplifications	Page 7, table 3
Outcomes and prioritization	13	List and define all outcomes for which data will be sought, including prioritization of main and additional outcomes, with rationale	Table 3
Risk of bias in individual studies	14	Describe anticipated methods for assessing risk of bias of individual studies, including whether this will be done at the outcome or study level, or both; state how this information will be used in data synthesis	Page 7, AMSTAR
Data synthesis	15a	Describe criteria under which study data will be quantitatively synthesised	N/A
	15b	If data are appropriate for quantitative synthesis, describe planned summary measures, methods of handling data and methods of combining data from studies, including any planned exploration of consistency (such as I^2, Kendall's τ)	N/A
	15c	Describe any proposed additional analyses (such as sensitivity or subgroup analyses, meta-regression)	N/A
	15d	If quantitative synthesis is not appropriate, describe the type of summary planned	
Meta-bias(es)	16	Specify any planned assessment of meta-bias(es) (such as publication bias across studies, selective reporting within studies)	The results of each included review paper will not be independently reported as this study is not being conducted within a systematic review methodology. A brief synthesis of overall study findings will be

For peer review only

			reported narratively.
Confidence in cumulative evidence	17	Describe how the strength of the body of evidence will be assessed (such as GRADE)	Page 9, GRADE

*** It is strongly recommended that this checklist be read in conjunction with the PRISMA-P Explanation and Elaboration (cite when available) for important clarification on the items. Amendments to a review protocol should be tracked and dated. The copyright for PRISMA-P (including checklist) is held by the PRISMA-P Group and is distributed under a Creative Commons Attribution Licence 4.0.**

From: Shamseer L, Moher D, Clarke M, Ghersi D, Liberati A, Petticrew M, Shekelle P, Stewart L, PRISMA-P Group. Preferred reporting items for systematic review and meta-analysis protocols (PRISMA-P) 2015: elaboration and explanation. BMJ. 2015 Jan 2;349(jan02 1):g7647.

For peer review only
